# Constitutive Model for Equivalent Stress-Plastic Strain Curves Including Full-Range Strain Hardening Behavior of High-Strength Steel at Elevated Temperatures

**DOI:** 10.3390/ma15228075

**Published:** 2022-11-15

**Authors:** Xiang Zeng, Wanbo Wu, Juan Zou, Mohamed Elchalakani

**Affiliations:** 1 School of Civil Engineering and Architecture, Hainan University, No. 58 Renmin Avenue, Haikou 570228, China; 2 College of Urban Construction, Hainan Vocational University of Science and Technology, No. 18 Qiongshan Avenue, Haikou 571137, China; 3 The Department of Civil, Environmental and Mining Engineering, Faculty of Engineering, Computing and Mathematics, The University of Western Australia, 35 Stirling Hwy, Crawley, Perth, WA 6009, Australia

**Keywords:** high-strength steel, elevated temperature, equivalent stress-plastic strain curves, necking, inverse finite element analysis, constitutive model

## Abstract

High-strength steel has been increasingly applied to engineering structures and inevitably faces fire risks. The equivalent stress-plastic strain ($\sigma_{\mathrm{eq}}$− $\varepsilon_{\mathrm{eqp}}$) curves of steel at elevated temperatures are indispensable if a refined finite element model is used to investigate the response of steel members and structures under fire. If the tensile deformation of steel is considerable, the $\sigma_{\mathrm{eq}}$− $\varepsilon_{\mathrm{eqp}}$ curves at elevated temperatures are required to consider the strain-hardening behavior during the post-necking phase. However, there is little research on the topic. Based on the engineering stress-strain curves of Q890 high-strength steel in a uniaxial tension experiment at elevated temperatures, the $\sigma_{\mathrm{eq}}-\varepsilon_{\mathrm{eqp}}$ curves before necking are determined using theoretical formulations. An inverse method based on finite element analysis is used to determine the $\sigma_{\mathrm{eq}}$− $\varepsilon_{\mathrm{eqp}}$ curves during the post-necking phase. The characteristics of $\sigma_{\mathrm{eq}}$−$\varepsilon_{\mathrm{eqp}}$ curves, including the full-range strain hardening behavior at different temperatures, are discussed. An equivalent stress-plastic strain model of Q890 steel at elevated temperature is proposed, which is consistent with the $\sigma_{\mathrm{eq}}-\varepsilon_{\mathrm{eqp}}$ curves. The constitutive model is further verified by comparing the finite element analysis and test results.

## 1. Introduction

Fire is one of the common disasters suffered by engineering structures. Under the high temperature caused by fire, the mechanical properties of steel deteriorate, and even the strength of steel gets lost. That may cause the loss of structural integrity or stability, severe structural damage, and even induce the collapse of structures [1,2]. Thus, evaluating the fire resistance of steel structures is significantly important, requiring knowledge of the mechanical properties of steel at elevated temperatures. The temperature-dependent mechanical behaviors of different structural steels have been studied [3,4,5,6,7,8,9,10,11,12,13,14,15,16,17,18,19,20]. Most of the literature reported the effect of temperature on the mechanical properties, including the elastic modulus [3,4,5,6,7,8,10,11,12,13,14,15,16,17,18,19,20], yield stress [3,4,5,6,7,8,10,11,13,14,15,16,17,18,19,20], ultimate tensile strength [3,4,5,6,7,10,11,13,15,17,18,19], ultimate strain at ultimate tensile strength [13] and fracture strain [5,6,13,18]. Different empirical formulas for the reduction factors of these mechanical properties at elevated temperatures were put forward through regression analysis based on the test data.

Due to the complexity of the mechanical behavior of steel structures under fire and the limit of experimental study (e.g., high cost and time consuming), numerical analysis models [1,2] have become an essential and effective means to investigate and evaluate mechanical behavior. It can also provide more response information than experiments. For numerical analysis models, the temperature-dependent stress-strain curves of steel are necessary, generally determined by the stress-strain model. Therefore, besides the above mechanical properties at elevated temperatures, the temperature-dependent stress-strain model is vitally significant for the fire-resistance assessment of steel structures. The existing temperature-dependent stress-strain models can be divided into engineering and true stress-strain models. Except for the NIST model [20], the other models belong to the engineering stress-strain model. The most common-used engineering stress-strain models of structural steel for the resistant-fire analysis are the EC3 model [21] and the ASCE model [22]. Other engineering models include the modified Ramberg-Osgood models [15,17], the Pho model [23], and the simplified and detailed engineering stress-strain models proposed by Lee et al. [9] for ASTM A992 steel.

A comparative study [24] shows that the accurate constitutive model is essential to capture the elastoplastic response of steel structures under fire. When exposed to fire, steel members may have large deformation, and steel in tension enters the post-necking phase. In this situation, it is necessary to incorporate the post-necking strain-hardening behavior of steel into the temperature-dependent stress-strain model to obtain accurate numerical simulation results. Particularly, it is practical and valuable to develop steel’s equivalent stress-strain model for full-range strain hardening behavior (containing the post-necking strain hardening behavior) at elevated temperatures, which is essential for the definition of plasticity data in the finite element (FE) model [25]. The above temperature-dependent constitutive models, except for the EC3 model [21] and the detailed model [9], don’t consider the necking behavior. Although the two engineering stress-strain models (the EC3 model [21] and the detailed model [9]) give the post-necking curves, the post-necking equivalent stress-strain curves can’t be obtained by conversion from the models due to the nonuniform strain and stress fields during the post-necking phase [26,27]. A literature survey shows little research on steel’s equivalent stress-strain curves and constitutive models with full-range strain hardening behavior at elevated temperatures, although they have been studied at room temperature [25,26,27,28,29,30] and under different strain-rate loading conditions [31,32,33].

High-strength structural steel, having a nominal yield stress (*f*_y_) not less than 460 N/mm^2^ [21,34], has been widely used in high-rise, long-span, bridge, and offshore structures due to many advantages brought by its application [35,36,37]. Q890 high-strength structure steel (*f*_y_ ≥ 890 N/mm^2^), having promising application prospects, has been investigated at elevated temperatures [14] and after exposure to fire [38]. However, the constitutive model for equivalent stress-plastic strain curves at different elevated temperatures has not been investigated, which is adverse to the FE analysis of Q890 steel structures under fire and its application to the scenario with fire risks.

The paper aims to develop a constitutive model for the equivalent stress-plastic strain relationship of Q890 high-strength steel at elevated temperatures, including the full-range strain hardening behavior. The full-range strain hardening behavior contains the necking behavior with increasing equivalent stress. The constitutive model can provide a basis for the structure’s fire safety evaluation using the FE model, especially for the FE analysis of large deformation with necking. The paper consists of the following sections. Tensile tests and the main test results of Q890 high-strength steel at elevated temperatures are described in Section 2. Details of the procedure for determining the equivalent stress-plastic strain curves of Q890 steel at high temperatures are presented in Section 3. The FE model of the tensile test used in the procedure is presented in Section 4. The characteristics of equivalent stress-plastic strain curves, including full-range strain hardening behavior of Q890 steel at elevated temperatures, are discussed, and the constitutive model for the curves is proposed and verified in Section 5. The conclusions are drawn in Section 6.

## 2. Summary of Tensile Tests of Q890 High Strength Steel at Elevated Temperatures

**Figure 1:**
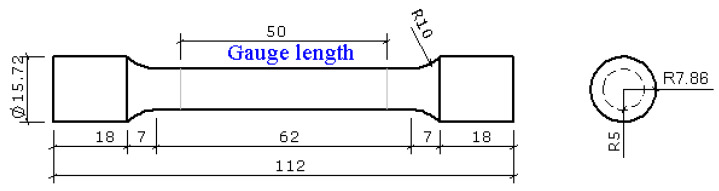
Geometry size of specimens (Unit: mm).

**Figure 2:**
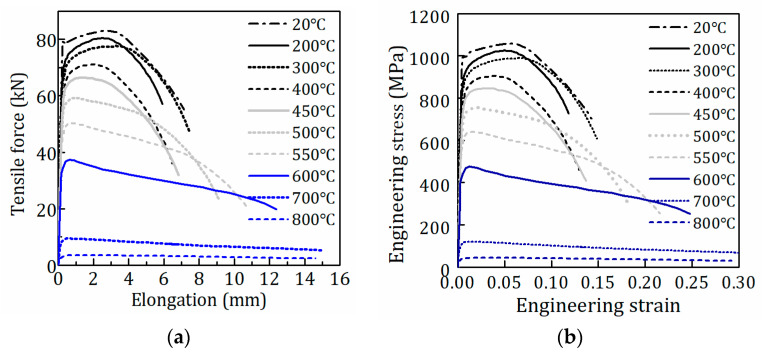
Test curves of Q890 steel at elevated temperature: (**a**) Tensile force-elongation curves; (**b**) Engineering stress-strain curves.

A series of uniaxial tensile tests have been performed on the round specimens of quenched and tempered Q890 high-strength steel at room temperature and elevated temperatures up to 800 °C by Huang et al. [14]. The geometry of round specimens is shown in Figure 1. The steady-state test method was used, and nine elevated temperature levels were set, including 200, 300, 400, 450, 500, 550, 600, 700, and 800 °C. In the test, the specimens were firstly heated to the target temperature with a heating rate of 10 °C/min. Then the target temperature was held for 15 min to acquire a uniform temperature field in the specimens. After that, specimens were stretched under the stationary temperature with a strain rate of 0.003/min until fracture. Figure 2 shows the tensile force-elongation curves and engineering stress-strain curves of Q890 steel at elevated temperatures, and Table 1 gives the mechanical properties. A detailed introduction of the experiment and test results can be found in [14].

## 3. Procedure for Determining the Equivalent Stress-Plastic Strain Curves of Q890 Steel at Elevated Temperatures

The equivalent stress-plastic strain curves are the input data to the FE model. According to the mechanical characteristics of tensile specimens, the engineering stress-strain ($\sigma_{n}–\varepsilon_{n}$) curves can be divided into the pre-necking and post-necking phases to acquire the equivalent stress-plastic strain curves of Q890 high-strength steel at elevated temperatures. Accordingly, the procedure for determining the curves is described in two sections.

### 3.1. Determination of Equivalent Stress-Plastic Strain Curves during the Pre-Necking Phase

It is well known that the necking of tensile specimens initiates at the ultimate tensile strength. In the pre-necking phase, the tensile specimen is under uniaxial uniform strain and stress in the gauge length. During this phase, the true stress ($\sigma_{\mathrm{true}}$), true strain ($\varepsilon_{\mathrm{true}}$) and true plastic strain ($\varepsilon_{\mathrm{tp}}$) can be obtained based on Equations (1)–(3). It is worth noting that the equivalent stress ($\sigma_{\mathrm{eq}}$) and strain ($\varepsilon_{\mathrm{eq}}$) are equal to the true stress and strain during the pre-necking phase [27]. Consequently, equivalent plastic strain $\left(\varepsilon_{\mathrm{eqp}}\right)$ is equal to the true plastic strain (Equation (3)). The $\sigma_{\mathrm{eq}}-\varepsilon_{\mathrm{eqp}}$ curves during the pre-necking phase can be calculated from the engineering stress-strain curves (Figure 2b) using Equations (1) and (3).

(1) $$\sigma_{\mathrm{eq}}=\sigma_{\mathrm{true}}=\left(1+\varepsilon_{n}\right)\sigma_{n}$$

(2) $$\varepsilon_{\mathrm{eq}}=\varepsilon_{\mathrm{true}}=\ln\left(1+\varepsilon_{n}\right)$$

(3) $$\varepsilon_{\mathrm{eqp}}=\varepsilon_{\mathrm{tp}}=\varepsilon -\frac{\varepsilon }{E_{T}}$$

### 3.2. Determination of Equivalent Stress-Plastic Strain Curves during the Post-Necking Phase

After necking, the stress status of tensile specimens is under complex stress conditions, and the stress is not uniform across the necking cross-section [27,28]. Equations (1)–(3) are no longer suitable during this phase. That makes it impossible to obtain the post-necking $\sigma_{\mathrm{eq}}-\varepsilon_{\mathrm{eqp}}$ curves directly from the engineering stress-strain response.

The Bridgman [28] and MLR [27] correction methods are usually used to obtain the post-necking strain hardening curves by correcting the average true stress corresponding to the instantaneous minimum sectional area during tension. The Bridgman correction method is inaccurate under large strain due to the assumption of uniform distribution of the equivalent strain in the minimum cross-section [25]. Moreover, it is expensive since the instantaneous curvature radius of the necking profile should be measured [25]. MLR correction method assumes that the ratio of equivalent stress to the average true stress is material-independent, including a regression equation for the ratio. Still, it needs to be verified when applied to structural steels [26] because the regression equation is based on limited test data. The diameter of the instantaneous minimum sectional area needs to be measured for the MLR correction method.

Recently, inverse methods based on the FE model have been widely used to identify steel’s hardening behavior after necking [25,29,30,31,32], providing accurate results and having a low cost. The method can modify the equivalent stress-strain curve by iterations, stopping when the FE analysis and test results are consistent. In different studies, test curves for comparison with FE analysis results are different due to the different shapes of specimens and measurement methods. Comparison of tensile force-elongation curves [29], tensile force-engineering strain curves [31,32], or engineering stress-strain curves [32] is the easiest means since only elongation in gauge length and tensile force should be measured in the tests. In this paper, the inverse method based on the FE model combined with the test data described in Section 2 is used to determine the $\sigma_{\mathrm{eq}}-\varepsilon_{\mathrm{eqp}}\;$curves during the post-necking phase.

The procedure for the inverse method based on the FE model to obtain $\sigma_{\mathrm{eq}}-\varepsilon_{\mathrm{eqp}}\;$curves are described as follows:

(1) Calculate the pre-necking $\sigma_{\mathrm{eq}}-\varepsilon_{\mathrm{eqp}}$ curves from the test curves with Equations (1)–(3), which is a part of the input data for the FE model described in Section 4.

(2) Establish an FE model to simulate the tensile tests of Q890 steel at different temperatures. The post-necking $\sigma_{\mathrm{eq}}-\varepsilon_{\mathrm{epq}}$ curves as the input data for the FE model is determined with the following two approaches.

Approach I—Iteration procedure for strain-hardening functions

(i) Use an appropriate strain-hardening function to extrapolate the post-necking $\sigma_{\mathrm{eq}}-\varepsilon_{\mathrm{eqp}}$ curves. Here, the Voce model (Equation (4)) [39] and Ludwik model (Equation (5)) [40] are adopted after different strain-hardening functions for steel are tried out.

(4) $$\mathrm{Voce}\;\mathrm{model}:\;\sigma_{\mathrm{eq}}=\sigma_{s}-(\sigma_{s}-\sigma_{i})e^{-n\varepsilon_{\mathrm{eqp}}}$$

where *n* is the material coefficient determining the development of strain hardening; $\sigma_{s}$ and $\sigma_{i}$ are the stress at saturation and the initial yield stress, respectively.

(5) $$\mathrm{Ludwik}\;\mathrm{model}:\;\sigma_{\mathrm{eq}}=\sigma_{0}+K_{L}\varepsilon_{\mathrm{eqp}}{}^{n}$$

where $\sigma_{0}$ is the initial yield stress of the material; $K_{L}$ is the hardening coefficient; *n* is the strain-hardening exponent.

According to the Considéré criterion, the strain-hardening functions (Equations (4) and (5)) satisfy Equation (6) at the onset of necking (the peak point of engineering stress-strain curves). The strain-hardening functions also satisfy Equation (7) since it passes the onset of necking. The parameters $\sigma_{s}$ and $\sigma_{i}\;$in Equation (4) and $\sigma_{0}$ and $K_{L}$ in Equation (5) can be determined using Equations (6) and (7) by assuming the *n* value. In other words, the two strain-hardening functions are definite for a given *n* value. In the analysis, an initial *n* value is assumed first, and the corresponding Voce curve or Ludwik curve is taken as the input data for the FE model.

(6) $${\left.\frac{d\sigma_{\mathrm{eq}}}{d\varepsilon_{\mathrm{eqp}}}\right|}_{\varepsilon_{\mathrm{eqp}}=\varepsilon_{\mathrm{tp}}^{u}}=\sigma_{\mathrm{true}}^{u}$$

(7) $${\left.\sigma_{\mathrm{eq}}\right|}_{\varepsilon_{\mathrm{eqp}}=\varepsilon_{\mathrm{tp}}^{u}}=\sigma_{\mathrm{true}}^{u}$$

where $\sigma_{\mathrm{true}}^{u}\;$and $\varepsilon_{\mathrm{tp}}^{u}$ are the true stress and plastic strain at the onset of necking.

(ii) Conduct FE analysis based on the acquired $\sigma_{\mathrm{eq}}-\varepsilon_{\mathrm{eqp}}$ curve and then get the tensile force-engineering strain curve.

(iii) Compare the tensile force-engineering strain curves obtained from the FE analysis and test. If the FE analysis curve is well consistent with the test curve, the corresponding $\sigma_{\mathrm{eq}}-\varepsilon_{\mathrm{eqp}}\;$curve is the desired curve. When the FE analysis curve doesn’t match the test curve, set a new *n* value and conduct a new FE analysis again. Through iterations, the optimum *n* value and $\sigma_{\mathrm{eq}}-\varepsilon_{\mathrm{eqp}}\;$curve can be obtained, which assures the consistency between FE analysis and test results.

**Figure 3:**
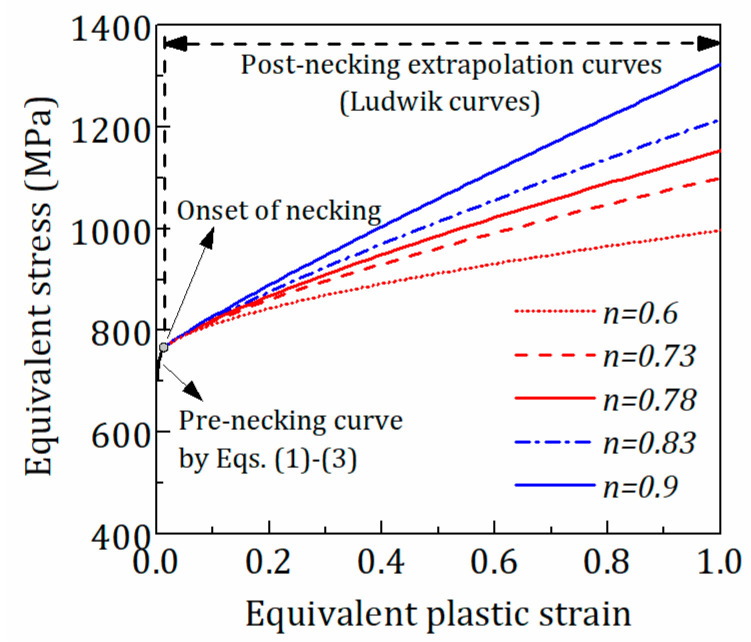
Ludwik curves with different *n* values at 500 °C.

**Figure 4:**
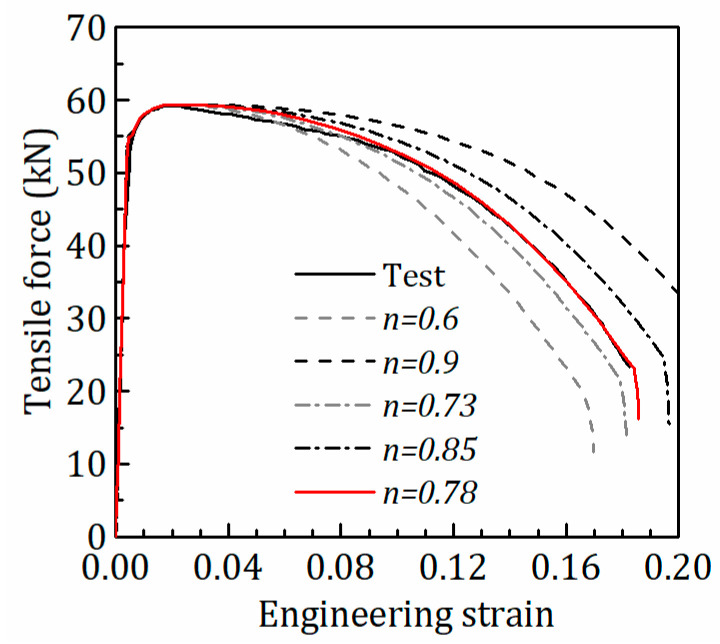
Comparison of FE analysis results from different Ludwik curves with test results at 500 °C.

As an example, Figure 3 shows the $\sigma_{\mathrm{eq}}-\varepsilon_{\mathrm{epq}}$ curves by extrapolation with the Ludwik model with different *n* values at 500 °C. Figure 4 shows the tensile force-engineering strain curves from FE analysis with the Ludwik model with different *n* at 500 °C after iteration. It is seen that the $\sigma_{\mathrm{eq}}-\varepsilon_{\mathrm{eqp}}$ curve corresponding to *n =* 0.78 can be used to simulate the test response accurately. In Table 2, the strain-hardening models with parameter values for the post-necking $\sigma_{\mathrm{eq}}-\varepsilon_{\mathrm{eqp}}\;$curves at 20–500 °C are tabulated.

In the temperature range between 550 and 800 °C, the authors didn’t find an appropriate strain-hardening function to describe the post-necking $\sigma_{\mathrm{eq}}-\varepsilon_{\mathrm{eqp}}\;$curves. Therefore, approach II was employed to determine the post-necking $\sigma_{\mathrm{eq}}-\varepsilon_{\mathrm{eqp}}\;$curves in this temperature range.

**Figure 5:**
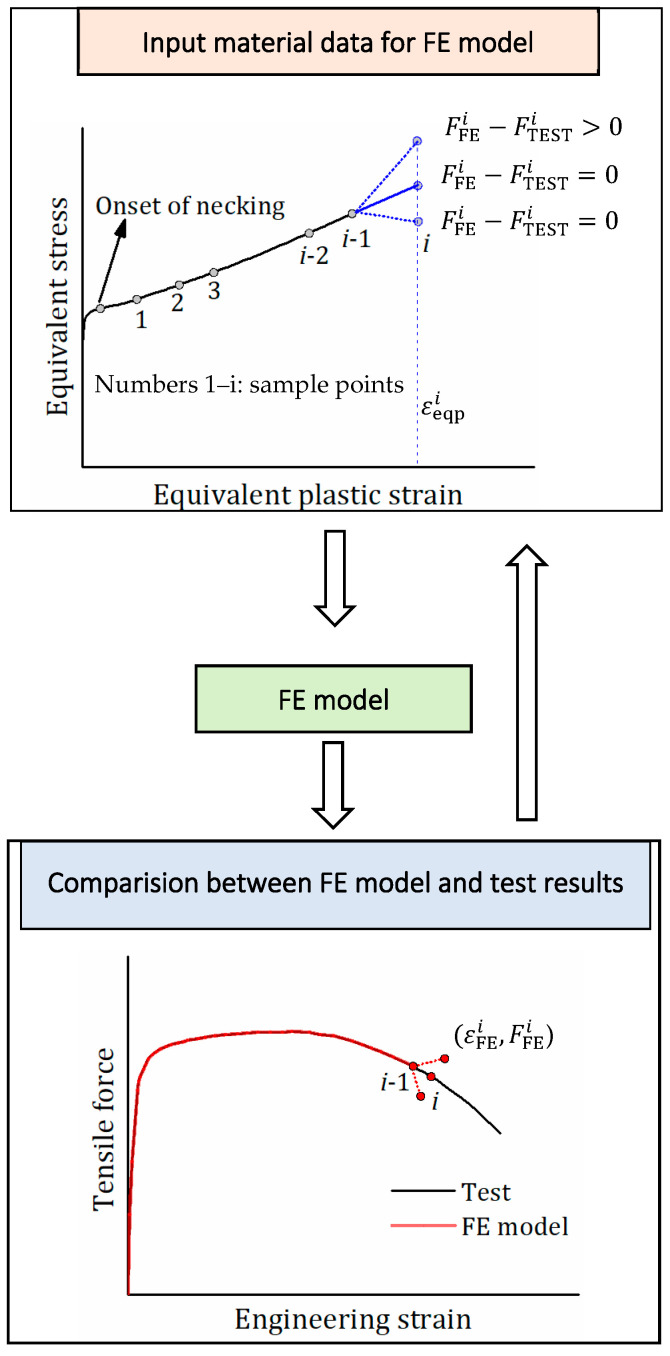
Diagrammatic sketch of the procedure for determining equivalent stress-plastic strain curves at 550–800 °C.

Approach II—Iteration procedure for equivalent stress (Figure 5)

(i) A series of sample points of equivalent plastic strain ($\varepsilon_{\mathrm{eqp}}^{i}$) during the post-necking phase are chosen. The sample point is with an interval of 0.01 to consider the apparent bending of the initial descending curves when the equivalent plastic strain is less than 0.1. When the equivalent plastic strain exceeds 0.1, the sample point is with a maximum interval of 0.2, which is turned down if the calculated curve deviates from the test curve due to the excessive strain interval. The equivalent stress $\sigma_{\mathrm{eq}}^{i}\;$at $\varepsilon_{\mathrm{eqp}}^{i}$ (initially *i* = 1) is assumed.

(ii) FE analysis is performed with the equivalent stress-plastic strain curve, including the pre-necking and post-necking curves. The post-necking curve is the piece-wise curve ($\varepsilon_{\mathrm{eqp}}^{k},\sigma_{\mathrm{eq}}^{k}$) (*k* = 1 to *i*). The tensile force-engineering strain curve is obtained from FE analysis, and the endpoint of the curve is ($\varepsilon_{\mathrm{FE}}^{i},\;F_{\mathrm{FE}}^{i}$), where $\varepsilon_{\mathrm{FE}}^{i}$ and $F_{\mathrm{FE}}^{i}$ are engineering strain and tensile force from FE analysis.

(iii) The FE analysis result is compared with the test curve. If the endpoint ($\varepsilon_{\mathrm{FE}}^{i},\;F_{\mathrm{FE}}^{i}$) does not fall into the test curve, the equivalent stress $\sigma_{\mathrm{eq}}^{i}$ at $\varepsilon_{\mathrm{eqp}}^{i}\;$is modified, and FE analysis is carried out again using the modified equivalent stress-plastic strain curve. The iteration process continues until the endpoint ($\varepsilon_{\mathrm{FE}}^{i},\;F_{\mathrm{FE}}^{i}$) is located on the test curve.

(iv) Repeat steps i–iii for the next sampling point $\varepsilon_{\mathrm{eqp}}^{i+1}$. In this step, the post-necking equivalent stress-plastic strain curve is unchanged from point 1 to *i*, and only the equivalent stress $\sigma_{\mathrm{eq}}^{i+1}$ is modified by iteration until the simulated curve is consistent with the test curve.

## 4. Description of FE Model

**Figure 6:**
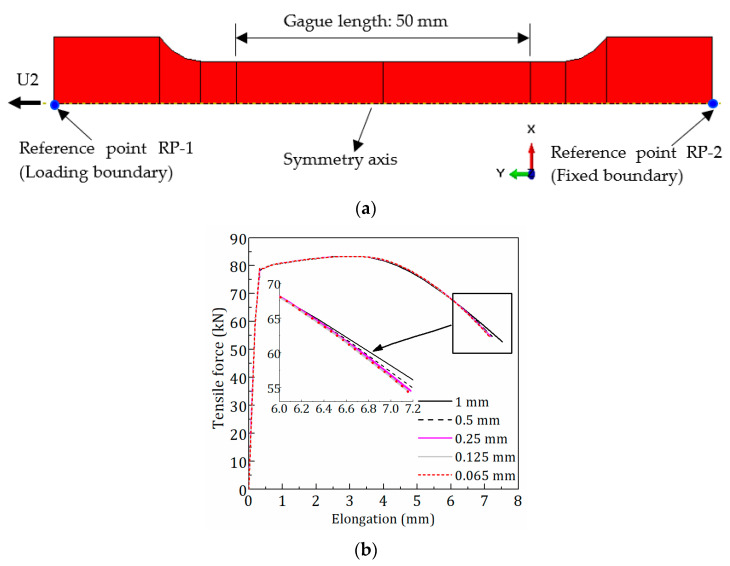

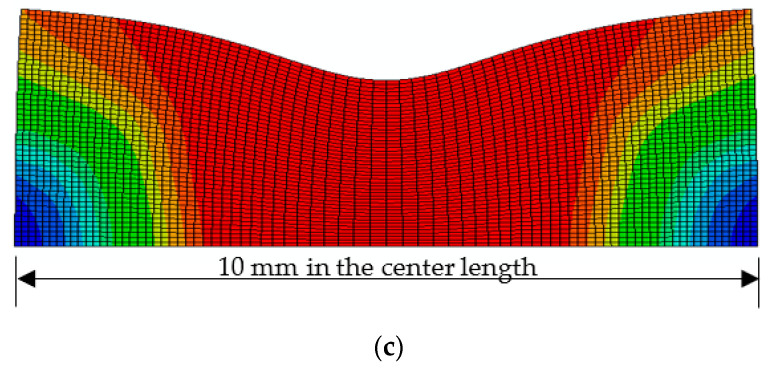
Axisymmetric finite element model of uniaxial tensile tests: (**a**) Boundary conditions; (**b**) Mesh convergence analysis; (**c**) Mesh (size: 0.125 mm).

The FE model was established based on the general-purpose software ABAQUS to complete the numerical analysis task. As shown in Figure 6a, an axisymmetric model for round specimens was employed to simplify the FE model and save computational costs.

The two end surfaces of specimens were coupled to reference points RP-1 and RP-2, respectively, with the kinematic coupling constraint. The fixed end was applied to RP-2 and the tensile displacement loading to RP-1.

The classical metal plasticity model with isotropic hardening in the ABAQUS material library was chosen to describe the behavior of steel. The $\sigma_{\mathrm{eq}}-\varepsilon_{\mathrm{eq}}$ curve obtained according to Section 3 is the necessary input data for the plasticity model. The fracture is simulated when the plastic strain reaches a critical value. The $\sigma_{\mathrm{eq}}$ is incremental with the $\varepsilon_{\mathrm{eq}}$ except for the last point of the $\sigma_{\mathrm{eq}}-\varepsilon_{\mathrm{eq}}$ curve. The last point is set artificially with equivalent stress close to zero (greater than zero to avoid the numerical difficulty) and equivalent plastic strain close to that of the penultimate point of the curve. That makes the $\sigma_{\mathrm{eq}}-\varepsilon_{\mathrm{eq}}$ curve drops sharply at the penultimate point, and the equivalent plastic strain of the penultimate point is the strain at fracture (critical value). The equivalent plastic strain at fracture is determined by a trial-and-error method until the endpoints of the force-engineering strain curve are consistent between numerical calculation and the tensile test.

Axisymmetric element CAX4R was used in the FE model, which is the 4-node, bilinear axisymmetric quadrilateral and reduced integration element. A sufficiently refined mesh is vital to yield accurate results. Therefore, a mesh convergence study was performed to determine the appropriate mesh size. As shown in Figure 6b, five approximate global mesh sizes (1 mm, 0.5 mm, 0.25 mm, 0.125 mm, and 0.065 mm) were used in the study. The analysis results suggested that it is rational to set the approximate global size to 0.125 mm, which makes the FE result converge well. Figure 6c shows the mesh size of 0.125 mm and the necking profile before fracture.

## 5. Constitutive Model for Equivalent Stress-Plastic Strain Curves of Q890 Steel at Different Temperatures

**Figure 7:**
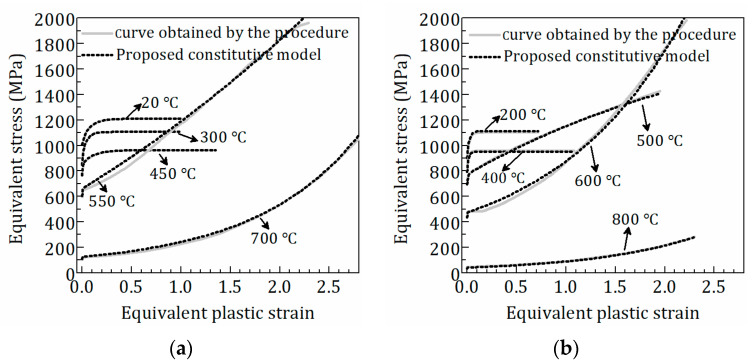
Comparison of the proposed constitutive model and equivalent stress-plastic strain curves: (**a**) 20, 300, 450, 550, and 700 °C; (**b**) 200, 400, 500, 600, and 800 °C.

The equivalent stress-plastic strain ($\sigma_{\mathrm{eq}}-\varepsilon_{\mathrm{eqp}}$) curves including full-range strain hardening behavior of Q890 steel at different temperatures are shown in Figure 7, which are obtained based on the above procedure. Figure 7 shows that the equivalent plastic strain at fracture differs at various temperatures, which is given in Table 3. The observation of the failure models [14] shows that the length of the necked region of the coupon increases at higher temperatures (500–800 °C), which is also reflected in the engineering stress-strain curves (Figure 2b) with larger strain at fracture. That means more reduction in sectional area and increasing equivalent strain at fracture at higher temperatures, which is consistent with the values in Table 3. Figure 2b shows that the post-necking phase of engineering stress-strain curves becomes less steep in the temperature range of 500–800 °C. Correspondingly, Figure 7 shows the notable change in the trend of equivalent stress-plastic strain curves at 450–550 °C. The $\sigma_{\mathrm{eq}}-\varepsilon_{\mathrm{eqp}}$ curves reach saturation stress at temperatures ranging from 20 to 450 °C. When Q890 steel is exposed to 500–800 °C, the $\sigma_{\mathrm{eq}}-\varepsilon_{\mathrm{eqp}}$ curves monotonically increase with equivalent plastic strain. The curves at 20–500 °C are characterized by the convex curve. On the contrary, the curves at 550–800 °C consist of a convex curve at the initial stage and a next concave curve. Therefore, different formulations are required to describe the $\sigma_{\mathrm{eq}}-\varepsilon_{\mathrm{eqp}}$ curves due to their discrepancy at different temperature ranges.

Although the calibrated formulations based on the NIST model for elastic modulus $\left(E_{T}\right)\;$and 0.2% proof stress $\left(f_{y,\;T}\right)\;$(engineering stress) of Q890 steel at elevated temperatures were provided by Huang et al. [14], equations (8) and (9) are proposed to more accurately predict the $E_{T}$ and $f_{y,\;T}$, respectively. Table 4 shows the comparison of the predicted results between Huang’s and present formulations for $E_{T}$ and $f_{y,\;T}$. It is seen that a significant error exists at 800 °C in the predicted results by Huang’s formulations and the present formulations have a slight error at different temperatures. Moreover, the current formulations have a low standard deviation, which means the scatter of the predicted results is slight.

(8) $$\frac{E_{T}}{E_{20}}=\frac{0.9828}{1+\exp\left[0.01237\left(T-639.5\right)\right]}$$

(9) $$\frac{f_{y,\;T}}{f_{y,20}}=\left\{\begin{array}{cc} 1.0111-5.56\times 10^{-4}\times T & 20\;{}^{\circ}C<T\leq 450\;{}^{\circ}C\\ 0.02376+0.7189/\left[1+\exp\left(T-612\right)/43.9\right] & 450\;{}^{\circ}C<T\leq 800\;{}^{\circ}C \end{array}\right.$$

where$\;E_{20}$ and$\;f_{y,20}$ are the elastic modulus and 0.2% proof stress at room temperature, respectively.

Through regression analysis, the constitutive model for the $\sigma_{\mathrm{eq}}-\varepsilon_{\mathrm{eqp}}$ curves is proposed as follows:

(10) $$\mathrm{For}\;20\;{}^{\circ}C\leq T\leq 500\;{}^{\circ}C,\;\sigma_{\mathrm{eq}}=\sigma_{\mathrm{eqy},\;T}+f_{H,\;T}\left(\varepsilon_{\mathrm{eqp}}\right)=\sigma_{\mathrm{eqy},\;T}+A_{1}\exp\left(k_{1}\varepsilon_{\mathrm{eqp}}\right)+A_{2}\exp\left(k_{2}\varepsilon_{\mathrm{eqp}}\right)+A_{0}$$

(11) $$\sigma_{\mathrm{eqy},\;T}=f_{y,\;T}(1+f_{y,\;T}/E_{T})\;$$

where$\;\sigma_{\mathrm{eqy},\;T}$ is the equivalent stress at initial yielding at *T* °C and Equation (11) is inferred from Equation (1) by assuming the linear elasticity behavior before initial yielding;$\;f_{H,\;T}$ represents the strain-hardening function at *T* °C. The values of the parameters in Equation (10) are listed in Table 5.

(12) $$\mathrm{For}\;500\;{}^{\circ}C<T\leq 800\;{}^{\circ}C,\;\sigma_{\mathrm{eq}}=\left\{\begin{array}{cc} \sigma_{\mathrm{eqy},\;T}+f_{H1,\;T}\left(\varepsilon_{\mathrm{eqp}}\right) & \varepsilon_{\mathrm{eqp}}\leq 0.01\\ \sigma_{\mathrm{eq},0.01}+f_{H2,\;T}\left(\varepsilon_{\mathrm{eqp}}\right) & 0.01<\varepsilon_{\mathrm{eqp}} \end{array}\right.$$

(13) $$f_{H1,\;T}\left(\varepsilon_{\mathrm{eqp}}\right)=B_{2}+\frac{B_{1}-B_{2}}{1+\exp\left(\varepsilon_{\mathrm{eqp}}-C\right)/D}\;$$

(14) $$f_{H2,\;T}\left(\varepsilon_{\mathrm{eqp}}\right)=a\left[1-\exp\left(-b\left(\varepsilon_{\mathrm{eqp}}-0.01\right)\right)\right]$$

where$\;\sigma_{\mathrm{eq},0.01}$ is the equivalent stress at $\varepsilon_{\mathrm{eqp}}=$0.01 at *T* °C;$\;f_{H1,\;T}$ and $f_{H2,\;T}$ represent the strain-hardening functions at different stages of the curve. The values of the parameters in Equations (13) and (14) are listed in Table 6.

**Figure 8:**
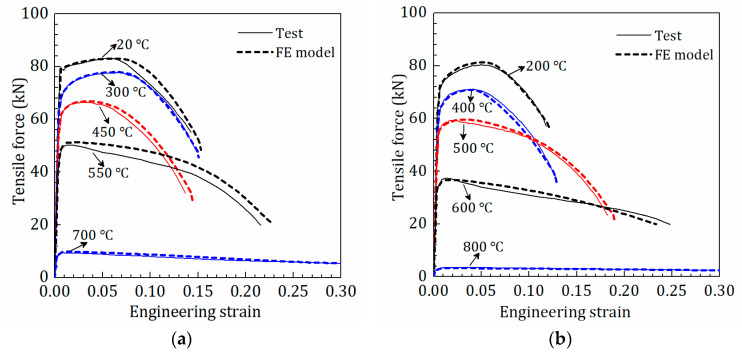
Verification of the proposed constitutive model: (**a**) 20, 300, 450, 550, and 700 °C; (**b**) 200, 400, 500, 600, and 800 °C.

In Figure 7, the comparison of the proposed constitutive model and the $\sigma_{\mathrm{eq}}-\varepsilon_{\mathrm{eqp}}$ curves obtained by the proposed procedure indicate that the proposed model is well consistent with the $\sigma_{\mathrm{eq}}-\varepsilon_{\mathrm{eqp}}$ curves. Moreover, the proposed model was used for the FE analysis of the tensile test of Q890 steel at elevated temperatures. As shown in Figure 8, the tensile force-engineering strain curves from the FE model agree with the test curves well, which furtherly verifies the validity of the proposed constitutive model.

## 6. Conclusions

In the paper, a constitutive model for equivalent stress-plastic strain ($\sigma_{\mathrm{eq}}-\varepsilon_{\mathrm{eqp}}$) curves of Q890 high-strength steel at elevated temperatures was investigated to provide a basis for the finite element analysis of the fire-resistant behavior of steel members and structures. The constitutive model includes the full-range strain hardening behavior, containing the post-necking behavior. A procedure was proposed for determining the $\sigma_{\mathrm{eq}}-\varepsilon_{\mathrm{eqp}}$ curves of Q890 steel at elevated temperatures from 20 to 800 °C. As a part of the procedure, an inverse method based on the finite element analysis was utilized to obtain the post-necking equivalent stress-plastic strain curves. Two iteration approaches for the inverse method were used in two different temperature ranges, respectively. The procedure successfully obtained the $\sigma_{\mathrm{eq}}-\varepsilon_{\mathrm{eqp}}$ curves.

Different trends of $\sigma_{\mathrm{eq}}-\varepsilon_{\mathrm{eqp}}$ curves are found in different temperature ranges: at elevated temperatures from 20 to 450 °C, the equivalent stress approaches saturation stress at large strain; at high temperatures not less than 500 °C, the equivalent stress monotonically increases with the increasing strain. The $\sigma_{\mathrm{eq}}-\varepsilon_{\mathrm{eqp}}$ curves are convex at elevated temperatures not exceeding 500 °C, but they become concave when the temperature reaches 550 °C.

Two formulations were proposed for elastic modulus and 0.2% proof stress of Q890 steel at elevated temperatures, which predicts the results more precisely than the existing formulations.

A constitutive model containing a series of formulations was proposed to describe the $\sigma_{\mathrm{eq}}-\varepsilon_{\mathrm{eqp}}$ curves including full-range strain hardening behavior of Q890 steel at 20–800 °C. The values of the parameters in the model were determined by regression analysis. The proposed constitutive model was verified by comparing the finite element analysis and test results.

## Figures and Tables

**Table 1:** Mechanical properties of Q890 high-strength steel at elevated temperature.

Temperature (°C)	Elastic Modulus *E*_T_ (MPa)	Yield Stress *f*_y,T_ (MPa)	Ultimate Tensile Strength *f*_u,T_ (MPa)
20	204,976	1003	1057
200	204,400	893	1025
300	201,032	847	990
400	183,367	798	906
450	175,592	761	845
500	174,246	698	754
550	150,692	593	640
600	132,509	441	475
700	60,189	104	120
800	23,209	37	45

Note: *f*_y,T_ means the 0.2% proof strength.

**Table 2:** Expressions of $\sigma_{\mathrm{eq}}-\varepsilon_{\mathrm{eqp}}\;$ curves during the post-necking phase at 20–500 °C.

*T* (°C)	Hardening Model	$\mathrm{Expression}:\;\sigma_{eq}$
20	Voce	$1211.3-163.5e^{-11\varepsilon_{\mathrm{eqp}}}$
200	Voce	$1103.4-155.1e^{-40\varepsilon_{\mathrm{eqp}}}$
300	Voce	$1107.5-168.6e^{-20\varepsilon_{\mathrm{eqp}}}$
400	Voce	$959.1-130.6e^{-60\varepsilon_{\mathrm{eqp}}}$
450	Voce	$963.1-115.9e^{-9\varepsilon_{\mathrm{eqp}}}$
500	Ludwik	$753.7-399.1\varepsilon_{\mathrm{eqp}}{}^{0.78}$

**Table 3:** Equivalent plastic strain at fracture of Q890 steel at elevated temperature.

*T* (°C)	20	200	300	400	450	500	550	600	700	800
$\varepsilon_{\mathrm{eqp}}\;$at fracture	1.006	0.719	0.985	1.08	1.35	1.95	2.29	2.22	2.92	2.3

**Table 4:** Comparison of predicted results between Huang’s and present formulations for $E_{T}$ and $f_{y,\;T}$.

*T* (°C)	20	200	300	400	450	500	550	600	700	800	Mean	Standard Deviation
$E_{T,1}/E_{T}$	1.000	0.990	0.984	1.029	1.029	0.967	1.003	0.964	1.120	0.749	0.984	0.0889
$E_{T,2}/E_{T}$	0.982	0.981	0.987	1.045	1.047	0.981	1.005	0.942	1.075	1.048	1.009	0.0397
$f_{y,\;T1}/f_{y,\;T}$	1.000	0.998	1.002	1.002	0.999	0.997	0.998	0.999	1.020	0.058	0.907	0.2832
$f_{y,\;T2}/f_{y,\;T}$	1.000	1.011	1.000	0.991	1.003	0.992	1.018	0.983	1.052	0.910	0.9960	0.0341

Note: the ratio in the first column means the ratio between predicted and test results; subscripts 1 and 2 represent Huang’s and the present formulations, respectively.

**Table 5:** Values of the parameters in Equation (10).

*T* (°C)	$A_{1}$	$A_{2}$	$A_{0}$	$k_{1}$	$k_{2}$
20	−87.62	−115.96	203.58	−24.10	−9.47
200	−14.39	−192.18	206.57	−136.43	−42.54
300	−81.13	−175.81	256.94	−123.61	−20.24
400	−128.7	−28.96	157.66	−82.17	−35.40
450	−89.1	−109.76	198.86	−137.55	−8.53
500	−1319.27	−78.33	1397.6	−0.34	−104.93

**Table 6:** Values of the parameters in Equations (13) and (14).

*T* (°C)	$B_{1}$	$B_{2}$	C	D	a	b
550	−3.9496	52.397	0.00327	9.53 × 10^−4^	−2682.4	−0.1809
600	−0.575	37.415	0.00394	6.82 × 10^−4^	−350.94	−0.764
700	−0.5068	16.9	0.00341	6.33 × 10^−4^	−72.98	−0.9435
800	−0.4473	7.606	0.00386	0.001	−30.6	−0.9442

## Data Availability

No new data were created or analyzed in this study. Data sharing is not applicable to this article.
